# The Generational Gift: The Effects of Grandparental Care on the Next Generations' Health and Well‐Being

**DOI:** 10.1002/hec.70054

**Published:** 2025-12-17

**Authors:** Mara Barschkett, C. Katharina Spiess, Elena Ziege

**Affiliations:** ^1^ IZA Bonn Bonn Germany; ^2^ University of Bonn Bonn Germany; ^3^ DIW Berlin Berlin Germany; ^4^ Federal Institute for Population Research (BiB) Wiesbaden Germany; ^5^ Johannes Gutenberg University Mainz Mainz Germany

**Keywords:** child health, grandparental child care, parental health, parental well‐being

## Abstract

Health and well‐being in the family context can be affected by care giving arrangements. Following parental care and daycare, grandparents are the third most important care givers for children in many Western societies. Despite the relevance of grandparental care, there is little evidence on the causal effects of this care mode on the next generations' health and well‐being. In this paper, we fill this gap by investigating the causal impact of regular grandparental care on the self‐reported health and (domain‐specific) satisfaction of both parents and children. To do so, we exploit geographic distance to grandparents as a source of arguably exogenous variation and use representative German panel data for families with children under the age of 11. Our results suggest positive effects on parental satisfaction with the child care situation, as well as mothers' satisfaction with their leisure time. However, we also find negative effects on children's health, particularly for elementary school aged children and for boys.

## 1 Introduction

Health and well‐being are central priorities for policymakers (see, e.g., Helliwell et al. 2024; OECD 2023a; UN 2022), as they are not only human rights but also important drivers of human capital accumulation (e.g., Currie 2020), labor force participation (e.g., Frijters et al. 2014), and, ultimately, economic growth (e.g., Well 2007). In addition to the individual level, health and well‐being are relevant study objects due to their intergenerational transmission and persistence (e.g., Coneus and Spiess 2012; Datta Gupta et al. 2023). Therefore, it is important to study health in the family context and the influence of interfamilial interactions. This provides a better understanding of how the larger family impacts the health of individual members, in this case children and their parents. One important interaction is caregiving, for example grandparents caring for their grandchildren. Grandparental care is the third most important care option, following parents and daycare, in many OECD countries (OECD 2019).^1^ However, the extent of grandparental care varies considerably due to country‐specific differences in daycare settings and female labor force participation. For instance, in Germany, a country with relatively low maternal employment rates and a universal daycare system, approximately one in four children under the age of 11 receives regular care from their grandparents (Section 2). Despite the expansion of daycare slots over the last decades in Germany, the importance of grandparental care has remained relatively stable over time (see Gambaro et al. 2024).

Despite the continuous importance of grandparents in providing child care, its effects on parental and child health and well‐being have received little attention in the literature.^2^ In this paper, we fill this research gap by adopting a double‐generation perspective and estimating the effects of grandparental care on parental and child health and well‐being.^3^ Identifying a causal relationship between grandparental care and these outcomes poses a challenge due to the endogeneity of the care decision. To overcome this, we utilize distance to grandparents as an instrumental variable (IV), assuming that the proximity to grandparents only affects parental and child outcomes through the provision of care. Using this instrument, one might have concerns regarding the validity of the exclusion restriction. The main threats to validity include differences between families living closer or further away from grandparents and strategic relocation patterns. We return to this issue in detail below, providing evidence that we can exclude major concerns regarding the exogeneity of the instrument. For example, we combine the IV approach with entropy balancing to ensure comparability between families residing close and far from grandparents and we show that our results are robust to relaxing the exclusion restriction. Additionally, we demonstrate that neither parents nor grandparents strategically relocate around the time of childbirth.

Our analysis is based on a sample of families with children below 11 using *pairfam*, a representative panel data set for Germany surveyed between 2009 and 2020. We consider outcomes at the parental and child levels. Specifically, we evaluate parents' subjective health and various dimensions of well‐being, including life satisfaction and domain‐specific satisfaction such as satisfaction with their leisure or the child care situation. For children, we use a parent‐assessed health measure. Underlying reasons why grandparental care may have effects on children and their parents can be manifold. In terms of parental outcomes, one hypothesis is that grandparental care increases parental satisfaction with leisure activities by providing parents with more time for activities unrelated to child care. A contrasting hypothesis is that grandparental care reduces health and well‐being of parents by increasing emotional stress between the grandparents and parents, as relationships within the family are prone to emotional conflicts and disagreements about child rearing (Clark et al. 2020). Additionally, grandparental care may be less stable or continuous compared to other forms of care due to the grandparents' potential illness^4^ or other obligations, which could result in higher levels of parental stress. Regarding child outcomes, we consider two competing hypotheses. The first hypothesis posits that grandparental care may negatively impact a child's health. Previous research has shown mixed results for (non‐)cognitive skills, with a slight tendency toward negative effects (e.g., Danzer et al. 2020; Zhang et al. 2021). Supporting this hypothesis, Ao et al. (2021) demonstrate that grandparents in China are less strict about limiting children's TV viewing time compared to parents, potentially reducing time spent on structured sports and outdoor activities, which could negatively affect the child's health. The opposing hypothesis suggests that grandparental care could have positive effects on the child's health. This hypothesis is based on the notion that grandparents may have more time to dedicate solely to the child, providing focused attention and care. In general, the intensity of grandparental care—even if provided regularly—may not be significant enough to substantially impact child and parent outcomes. Therefore, it remains an empirical question of whether grandparental care has implications for the next generations' health and well‐being.

Overall, our results provide evidence that, grandparental care is beneficial, particularly for maternal well‐being. We show that grandparental care increases maternal satisfaction with child care and leisure by 9 and 11%, respectively, compared to the mean. Furthermore, we find substantial increases in fathers' satisfaction with child care (19%). The effects on satisfaction with child care are mostly driven by parents with higher education. However, we do not find evidence that grandparental care affects parental health, life satisfaction or other domains of satisfaction. By contrast, our analysis shows that grandparental care has a negative impact on children's health. These effects are more pronounced for boys and might be explained by differences in the afternoon program organized by schools/daycare centers compared to grandparents.

While the effects of grandparental care on the grandparents themselves have been studied extensively, less attention has been given to the outcomes for parents and children.^5^ Therefore, our study makes three contributions. First, we contribute to the literature on the effects of various care modes, in this case grandparental care, on parental outcomes by studying the effects of grandparental care on parental health and well‐being. The existing literature on the effects of other care modes—particularly daycare—on parental outcomes is vast and primarily focuses on maternal employment (for an overview, see Müller and Wrohlich 2020), fertility (e.g., Bauernschuster et al. 2016), health (e.g., Barschkett and Bosque‐Mercader 2024; Herbst and Tekin 2014), maternal well‐being (e.g., Kröll and Borck 2013; Schmitz 2019) and the integration of migrant mothers (e.g., Gambaro et al. 2021). Research on grandparental care and parental outcomes has mainly centered on maternal employment and fertility, indicating that the availability of grandparents leads to an increase in maternal employment (Aparicio Fenoll 2020; Bratti et al. 2018; Compton and Pollak 2014; Kanji 2018) and a shift in the timing of fertility (e.g., Eibich and Siedler 2020). We contribute to this literature by considering well‐being outcomes and subjective health, thereby focusing on other aspects that may be affected by grandparental care. As discussed above and supported by related research, both positive and negative effects may be observed for the different outcomes, highlighting the importance of considering a broad range of outcome variables to get a more comprehensive understanding of the effects of grandparental care.

Second, we add to the literature on the impact of different modes of care on child outcomes, which has previously mostly focused on daycare or parental care (for studies in the German context, see e.g., Barschkett 2022; Cornelissen et al. 2018; Felfe and Lalive 2018). The current body of causal evidence on informal care's influence on children is limited and primarily centered around (non‐)cognitive skills. Comparing children cared for by grandparents with those attending formal child care, Del Boca et al. (2018) find a positive association between grandparental care and children's cognitive skills for children from more advantaged households, while observing a negative association for children from less advantaged households. In a comparison of children cared for by grandparents and those primarily cared for by their parents, Ao et al. (2021) find that children in grandparental care exhibit a greater external locus of control. Additionally, Zhang et al. (2021) report that these children demonstrate lower abilities in walking, talking, counting, and toilet training. Danzer et al. (2020) also show that care provided by mothers or formal institutions is superior to informal care arrangements regarding children's development. Furthermore, the study by Kaufmann et al. (2022) finds an increase in preschool children's test scores when using maternal care instead of grandparental care, alongside a decrease in the test scores of 11‐ to 12‐year‐old boys when switching from grandparental care to after‐school care. By contrast, evidence on the causal impact on children's health is limited.^6^ We contribute to this literature by providing evidence on the causal effects of grandparental care on child health. As discussed above, the empirical evidence on (non‐)cognitive skills suggests that this effect could operate in both directions and exhibit heterogeneity across groups.

**FIGURE 1:**
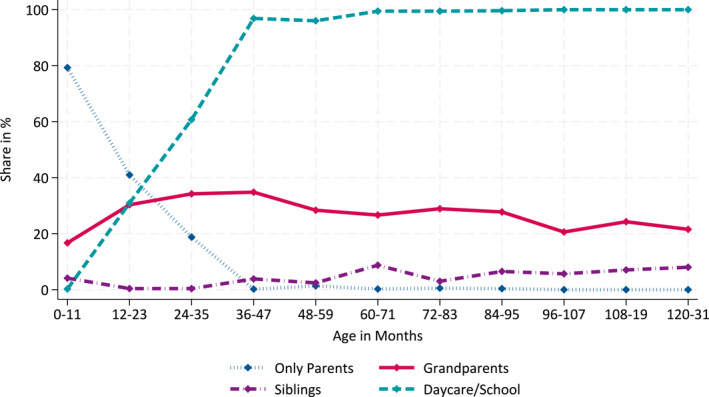
Actors and institutions involved in care of children younger than 11 in Germany. A child is counted as cared for by the grandparents in this graph if the child is cared for by its grandparents in the morning or afternoon or both. The same applies for the other actors. The category “only parents” is exclusive, as it includes only children not cared for by any other caregiver. The categories “grandparents,” “siblings,” and “daycare/school” are not mutually exclusive; a child can belong to one, two, or all three of these categories. *Source:* Pairfam (2018/19), weighted, own calculation.

Third, our study provides novel evidence on these specific outcomes for Germany, a context that offers valuable insights applicable to other countries. While existing literature has primarily focused on the United States or other European countries (for a summary see, e.g., Hank and Buber 2009), Germany presents a particularly interesting case for several reasons. Firstly, Germany is characterized by a highly subsidized universal daycare system that has expanded significantly over recent decades, mirroring trends in many other OECD countries. Secondly, despite this expansion of publicly funded child care, we demonstrate that approximately a quarter of children are still regularly cared for by their grandparents (Figure 1).^7^ A comparison of 26 European countries by Zanasi et al. (2023) shows that Germany's share of grandparental care is on par with the average of these countries. Thirdly, despite Germany's relatively low maternal full‐time and high part‐time employment rates compared to other EU countries (Eurostat 2023), Germany has seen a trend of defamilization over the last years (e.g., Zagel and Lohmann 2021). This unique combination of universal daycare, substantial grandparental involvement, and low full‐time maternal employment provides a distinct setting for studying the effects of grandparental care.

The remainder of this paper is structured as follows: In Section 2 we describe the institutional setting in Germany. Section 3 gives an overview of the data used. In Section 4 we present the empirical strategy. Section 5 reports the main findings, discusses the robustness of the results and presents the results of our mechanism analysis. Finally, Section 6 concludes the paper.

## 2 Institutional Setting

**FIGURE A1:**
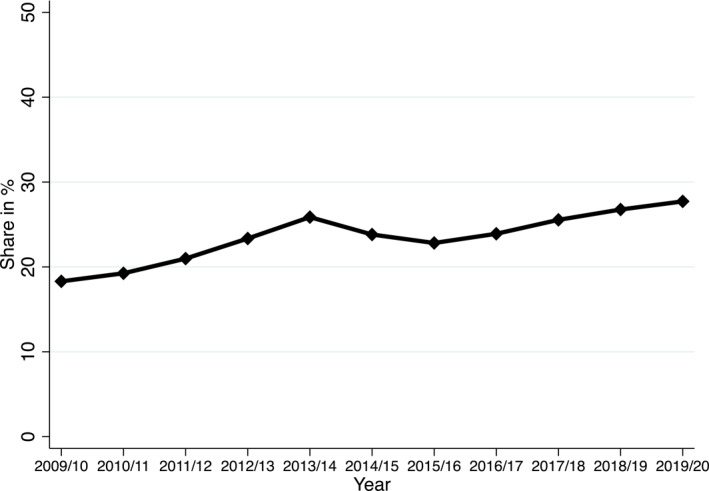
Development of grandparental care (2009–2020). The graph shows the development of grandparental care for children below the age of 6. A child is counted as cared for by the grandparents if the child is cared for by its grandparents in the morning or afternoon or both. *Source:* Pairfam (2009–2020), weighted, own calculation.

In Germany, regular grandparental care has played a significant role for many years (see Figure A1). Figure 1 demonstrates that in 2018/19, grandparents provided care for approximately 20–30% of children under the age of 11, across different age groups.

To understand the role of grandparental care in Germany and its evolution, it is important to consider other forms of child care that are also utilized, as well as trends in parental employment. Historically, Germany has had low rates of female employment, while full‐time employment for men has consistently been prevalent. However, in recent decades, there has been a notable increase in maternal employment in Germany. The percentage of working mothers has risen from 61.2% in 2006 to 73.8% in 2021 (e.g., OECD 2023b).^8^ This increase in maternal employment has been facilitated by a substantial expansion of publicly funded daycare since the 1990s (e.g., Müller and Wrohlich 2020). While enrollment in daycare for children above 3 years old has become almost universal (92% in 2022) since 2000 (Müller and Wrohlich 2020), about 70% of children are in full‐time care (Autorengruppe Bildungsberichterstattung 2020). Attendance rates for children below 3 years old are significantly lower but have increased from below 5% in 1990 to approximately 35.5% in 2022 (Statistisches Bundesamt 2022). Daycare fees in Germany are relatively low, and some states have even abolished them (e.g., Huebener et al. 2020; Schmitz et al. 2017). Most daycare centers in Germany are operated by non‐profit organizations or municipalities (Spiess 2008). However, during a child's first year, parents usually care for their child themselves while being on paid parental leave, which can last up to 14 months (see Figure 1). Other forms of regular child care that have experienced significant increases in usage in recent years are all‐day schools or after‐school care programs for elementary school children. The proportion of children up to age 12 in all‐day schools or related programs has increased from 9.8% in 2002/03 to 49.2% in 2022/23 (Bundesministerium für Bildung und Forschung, 2024).

**FIGURE 2:**
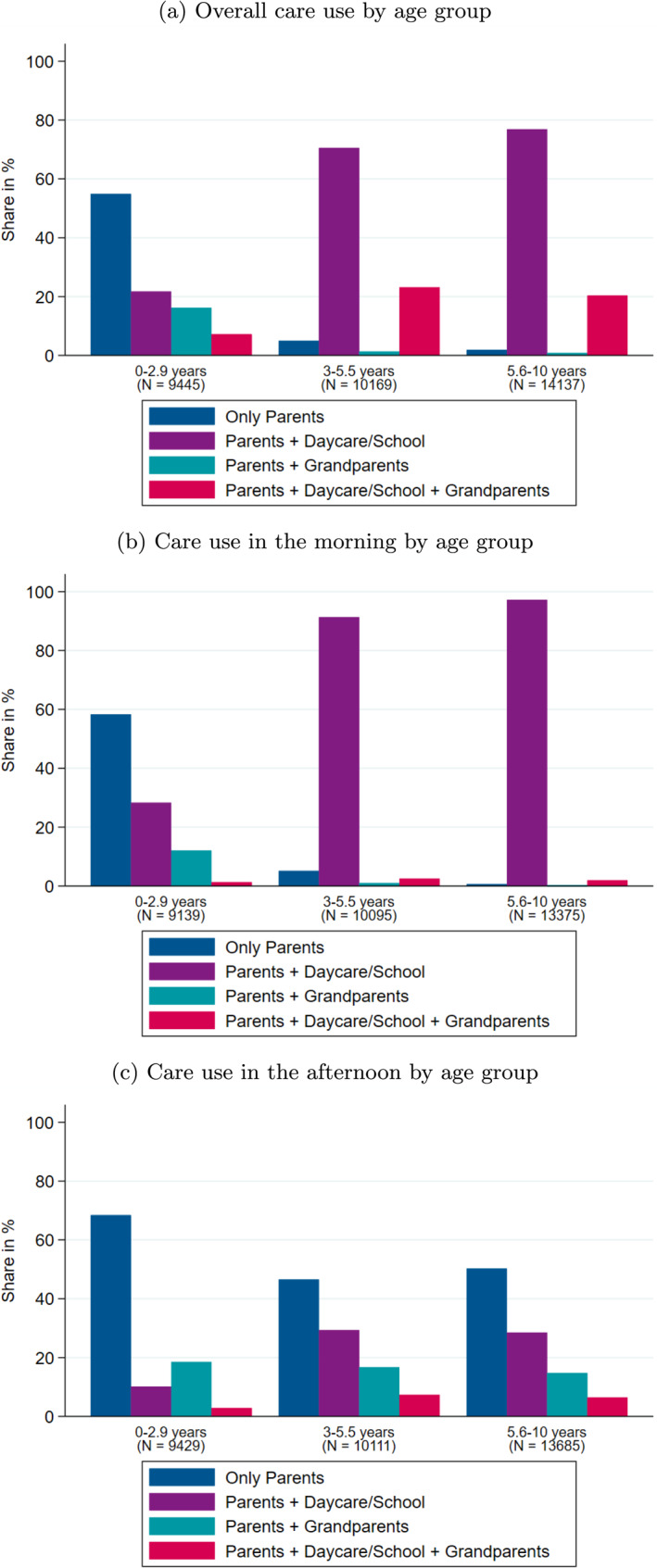
Care patterns. The figures show the care use by age group. Overall care use takes all actors either caring for the child in the morning or afternoon or both into consideration. *Source:* Pairfam (2009–2020), weighted, own calculation.

Next to formal care arrangements, grandparents play an important role in the “care puzzle” (see Gambaro et al. 2024). Figure 2 shows the distribution of various care modes for different age groups of children pooled from 2009 to 2020. The majority of young children (aged 0 to under 3 years) are primarily cared for by their parents. In the morning, the second most commonly used option is a combination of parental and daycare, which applies to approximately 25% of children. This is followed by a mix of parental and grandparental care, which accounts for about 15%. In the afternoon, the combination of parental and grandparental care is the second most frequently chosen option (20%), while only about 10% of children receive care from both parents and daycare. Therefore, for this age group, we define exclusive parental care as the alternative to grandparental care.

Older children (aged 3–5.5 years and 5.6–10 years) are predominantly cared for by a combination of parents and daycare/school (70–80%). However, there are significant differences between morning and afternoon arrangements: in the morning, 90–95% of children receive care from either daycare or school, whereas in the afternoon, the majority of children are exclusively cared for by their parents (around 50%). When parents do not provide exclusive care, it is observed that most families opt for a combination of parents and daycare/school (30%) or a combination of parents and grandparents (almost 20%). The least common option is a combination of daycare/school and grandparents, which is chosen by less than 10% of families in the afternoon. Consequently, it can be concluded that the most prevalent alternative to grandparental care for older children is either sole parental care or parental care combined with daycare or a school program in the afternoon.

## 3 Data

For the analysis, the “Panel Analysis of Intimate Relationships and Family Dynamics” (*pairfam*) dataset is utilized (Huinink et al. 2011). The participants in this study are surveyed on an annual basis, which allows us to examine variations both between and within individuals (Huinink et al. 2011). While Pairfam interviews all generations separately, our study relies exclusively on information provided by parents, who report on themselves, their children, and grandparents. Additional details about the dataset can be found in Appendix B.

### 3.1 Grandparental Care Variable

The main explanatory variable in our analysis is grandparental care. We have information on grandparental care for each child separately, both in the morning and afternoon. However, our data does not distinguish between grandmothers and grandfathers as caregivers. To conduct our analysis, we create a binary variable that indicates whether a child is regularly cared for by their grandparents in the morning, afternoon, or both, but we do not have information on the number of hours. In order to examine parental well‐being^9^ and health, we use a binary variable. This variable is set to one if at least one child of the parent in question is cared for by grandparents in the morning, afternoon, or both.^10^ The use of grandparental care within families remains relatively stable over time, so most of the variation in this variable comes from comparisons between different families.

### 3.2 Parental Health and Well‐Being

We analyze various variables related to subjective parental health and satisfaction in our study. The first variable, called health, is an ordinal variable that ranges from 1 (very bad health) to 5 (very good health). This self‐assessed, subjective health measure is an aggregate measure of various health dimensions including physical and mental health. To evaluate well‐being, we consider six satisfaction variables, all of which are ordinal variables measured on an 11‐point Likert scale ranging from 0 (very dissatisfied) to 10 (very satisfied). The first variable measures general satisfaction with life. Additionally, *pairfam* includes several variables capturing domain‐specific satisfaction, such as satisfaction with school, education, or career; satisfaction with leisure activities, hobbies, and interests; satisfaction with the relationship with one's partner; satisfaction with work‐life balance; and satisfaction with the child care situation for each child. All these variables have been surveyed annually since 2009, except for satisfaction with work‐life balance, which has been included only since 2013.

The table including our main results for parental outcomes (Table 1 in Section 5) includes the sample averages for all our outcome measures in column 4. In general, mothers and fathers report similar levels of health and satisfaction across most outcomes. Both groups perceive their health as good, with mothers averaging a score of 3.6 and fathers averaging 3.8. Moreover, individuals in our sample exhibit relatively high levels of satisfaction, ranging from 5.9 to 8.4. The sample sizes vary for different outcome variables as they are surveyed in different survey waves, with relationship satisfaction being surveyed only among individuals in a relationship and child care satisfaction measured at the child level. However, using a harmonized sample for outcomes surveyed in the same waves does not affect the results of our analysis.

### 3.3 Child Health

We also analyze the impact of grandparental care on the health of children. To measure this impact, we consider the assessment of children's health made by their mothers or fathers. Similar to parental health, this variable is ordinal, ranging from 1 (very bad health) to 5 (very good health). In Table 2 (Section 5), which reports our main regression results, column 4 displays the sample means for each age group, as well as the pooled mean across all age groups. On average, parents rate their children's health as very good, with a mean of 4.4. Interestingly, the perception of health is very similar across all three age groups.

### 3.4 Measurement of the Instrument

We use the distance to grandparents as an instrument to measure grandparental care, as explained in Section 4. In the *Pairfam* data, we have information about the geographical distance between the household and all four grandparents (if they are still alive).^11^ The distance is categorized into six categories.^12^ Based on this, we construct a binary variable that is equal to one if at least one grandparent lives closer than 30 min, and zero otherwise. We use this binary variable because the relationship between distance and the extent of grandparental care provided is unlikely to be linear. For example, the difference between living 10 or 30 min away should have a greater impact than the difference between 3 and 3 h and 20 min. We chose 30 min as the cutoff because it is a reasonable distance that still allows commuting within one day when providing care to a grandchild.^13^

**FIGURE A2:**
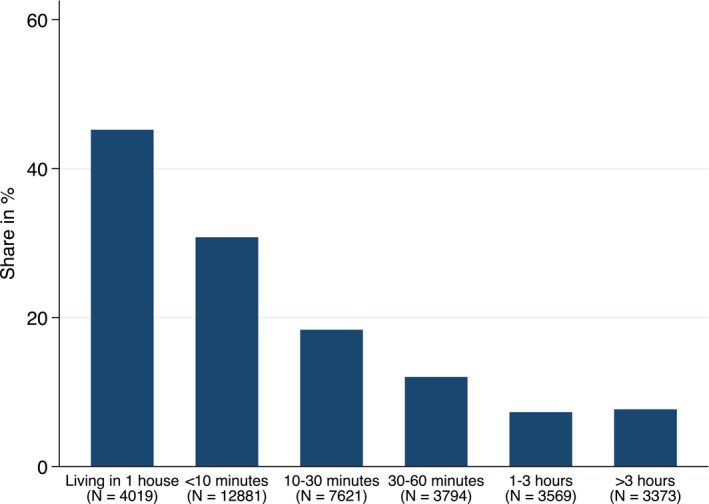
Grandparental care by distance. The figures show the share of children cared for by grandparents by the distance between the child's household and the closest living grandparent. A child is counted as cared for by the grandparents in this graph if the child is cared for by its grandparents in the morning or afternoon or both. *Source:* Pairfam (2009–2020), weighted, own calculation.

The distribution of the ordinal distance variable used to construct our instrument and the grandparental care variable can be seen in Figure A2. This figure shows the percentage of children in grandparental care based on the minimum distance between the child and the grandparents. In our sample, approximately 69% of households live less than 30 min away from at least one grandparent, indicating that most children live close to at least one grandparent.^14^ Additionally, we observe that the percentage of households using grandparental care increases non‐linearly as the distance decreases.^15^

### 3.5 Control Variables

To account for other observable factors that might confound the effect of grandparental care on the health and well‐being of both parents and children, our models include a comprehensive set of control variables at various levels: (grand‐)parental, child, and household. Specifically, we include socio‐economic characteristics of the parents, such as education, age, income, gender, federal state of residence, and migration background. Furthermore, we include detailed information about the household situation, such as the number of children in the household and the age of the youngest child. For a complete list of control variables for each outcome variable, please refer to Table A1.^16^ In order to ensure the robustness of our findings, we conduct additional tests where we vary the set of included control variables, such as excluding potentially endogenous variables like income, and demonstrate that our results remain consistent.

### 3.6 Samples

We conduct analyses at both the child and parent levels. To evaluate the effects on parental health and satisfaction, our analysis is limited to individuals who have at least one child. Each parent constitutes one observation. The analysis of parental satisfaction with child care and child health is conducted at the child level. Additionally, we only include families where at least one parent was born in Germany. If both parents were born outside of Germany, it is highly likely that none of the four grandparents lives in Germany, making regular child care unavailable (e.g., Gambaro et al. 2018). We observe the samples at the parental level and the child level from 2009 to 2020.^17^

Our final sample for analyzing parental outcomes consists of 16,056 observations for fathers (corresponding to 4043 fathers) and 19,844 observations for mothers (corresponding to 4788 mothers). The sample for analyzing child health includes 44,339 observations, corresponding to 11,714 children. Detailed summary statistics can be found in Table A2.

## 4 Empirical Strategy

Identifying a causal effect of grandparental care on parental health and satisfaction and child health faces potential endogeneity threats. The choice for grandparental care is endogenous. Thus, it may be influenced by unobserved characteristics that also influence the outcome variables, resulting in an omitted variable bias. One example of such an unobserved variable is a grandparent's preference for taking care of their grandchild. This preference likely affects the amount of support grandparents offer and may directly impact the outcomes we are interested in. Another threat is reverse causality, where parental well‐being may influence the assistance they need from grandparents and consequently the demand for grandparental care. Similarly, the health of children likely influences the decision to seek help from grandparents. For instance, parents with children who have poor health may worry that taking care of such children would be burdensome for grandparents, or they may rely on grandparents because other non‐parental care options are not feasible.

Therefore, estimating an ordinary least squares (OLS) specification may yield biased and inconsistent estimates of the effect of grandparental care, failing to capture any causal relationship. Both upward and downward biases in OLS estimators are possible. For example, if only healthy children receive grandparental care, the OLS estimator is expected to be upward biased. Conversely, if parents with low subjective well‐being are more likely to seek help from grandparents due to greater need for assistance, the OLS estimator would be downward biased.

To address the endogeneity problem, we employ an instrumental variable (IV) approach. We can use an instrument that determines the endogenous regressor $\left(GPC_{it}\right)$, but only affects the dependent variables $\left(y_{it}\right)$ through its effect on this independent variable (grandparental care). In this study, we use the distance to the grandparents as an instrument, which has also been utilized by Del Boca et al. (2018) and Compton and Pollak (2014).

In the first stage of our two‐stage least squares (2SLS) approach, we regress the grandparental care variable on our instrument and the exogenous control variables:

(1) $$GPC_{it}=\gamma_{1}+\gamma_{2}D_{it}+X_{it}^{\prime }\gamma_{4}+\varepsilon_{it}$$

Here $D_{it}$ equals one if the household lives less than 30 min away from at least one grandparent, and 0 otherwise. The variable of interest, grandparental care $\left(GPC_{it}\right)$, is a binary variable, and $X_{it}^{\prime }$ represents our vector of control variables (e.g., including year and state fixed effects), as shown in Table A1 and described in Section 3. The first stage regression is estimated using OLS. Since the dependent variable is binary, this corresponds to a linear probability model (LPM, see Appendix E).

In the second stage, the fitted values of the linear probability model from the first stage, denoted as ${\hat{GPC}}_{it}$, are included as the main explanatory variable:

(2) $$y_{it}=\beta_{1}+\beta_{2}{\hat{GPC}}_{it}+X_{it}^{\prime }\beta_{3}+\mu_{it}$$

In this regression, $y_{it}$ refers to different parental health, satisfaction, and child health outcomes described in Section 3.^18^ $X_{it}^{\prime }$ is again our vector of control variables, which remains the same as in the first stage regression. The standard errors $\mu_{it}$ are clustered at the household level for the regressions of parental satisfaction with the child care situation and child health.^19^

$\beta_{2}$ is our coefficient of interest, which represents the 2SLS estimator. By definition, it estimates the local average treatment effect (LATE). It measures the effect on the compliers, that is, those families whose utilization of grandparental care is (not) induced by a (large) small distance to the grandparents.

Our sample comprises three types of subjects based on their response to the instrumental variable: (i) always‐takers, (ii) never‐takers, and (iii) compliers.^20^ Observable always‐takers are families who utilize grandparental care despite living at a considerable distance from the grandparents. Conversely, observable never‐takers are individuals who do not use grandparental care even when living in close proximity to the grandparents. Compliers cannot be identified at the individual level because compliers with a large distance are indistinguishable from never‐takers with a large distance, and compliers with a small distance are observably identical to always‐takers with a small distance. To address this, we employ the method proposed by Marbach and Hangartner (2020). By subtracting the weighted covariate mean of observable always‐takers and never‐takers from the covariate mean of the entire sample, we can deduce the covariate mean for compliers.

Table A3 presents a comparison of covariate means and standard deviations across subject types. Our sample consists of 8% always‐takers, 69% never‐takers, and 23% compliers. While compliers share similarities with always‐takers and never‐takers in certain aspects, they differ significantly in others. For instance, complier mothers exhibit comparable rates of employment and high educational attainment to always‐takers, whereas never‐takers demonstrate substantially lower rates of employment and educational attainment. Additionally, on average complier grandparents are older than always‐takers and never‐takers, suggesting that—in line with previous research (e.g., Backhaus and Barslund 2021; Frimmel et al. 2020; Tanskanen et al. 2021)—particularly grandparents who are retired deliver care to their grandchildren. If grandparents in the complier group are, on average, older, this may suggest that they are also in poorer health. Consequently, if worse grandparental health negatively affects parents' well‐being, it may indicate that we are underestimating the impact on parental satisfaction. Conversely, this could mean that we are overestimating the negative effect on child health. However, an additional analysis controlling for grandparental health does not provide evidence for this. Given that the LATE measures the treatment effect specifically for compliers, these socioeconomic disparities among the three groups suggest that extending the results to always‐takers or never‐takers may not be appropriate.

For the distance to grandparents to be considered a valid instrument, it must meet several conditions. Of particular importance are the relevance and exogeneity assumptions of the instrument. Relevance means that the instrument must be sufficiently correlated with the endogenous regressor, grandparental care. Arguably, the distance to the grandparents satisfies the relevance condition as a shorter distance facilitates grandparental care. The correlation between the instrument and the endogenous regressor is shown in Figure A2 and tested in the first stage regression, where the endogenous variable is regressed on the instruments and the exogenous covariates (Table A4). The robust first stage F‐statistics displayed in the main regression tables in Section 5 are at least 67, but in most regressions, they far exceed this value. This strengthens our argument.^21^

The more critical assumption is the exogeneity assumption of the instrument, which requires that the instrument is not correlated with the error term and thus influences the outcome variable only through the endogenous regressor. It seems plausible that distance affects child health only through grandparental care. This relationship is less straightforward for parents, as for example healthier grandparents might be more likely to provide child care and grandparental health might also directly affect parents' health and satisfaction (and potentially even children's health). We provide evidence through several robustness checks that we are likely to isolate the effect of grandparental care on parental health and satisfaction and child health. In Section 5.3, we provide a detailed discussion on the validity of this instrumental variable approach, including the plausibility of the exogeneity assumption, as well as the robustness of our results.

## 5 Results

### 5.1 Main Results

#### 5.1.1 First Stage

We begin the discussion on the effects of grandparental care by focusing on the first‐stage effects. In all the specifications, the impact of distance on grandparental care is consistently significant and of similar magnitude (Table A4 in the Appendix). Living within a half‐hour distance from at least one grandparent increases the likelihood of receiving grandparental care by approximately 24% points (depending on the sample). This indicates that our instrument is highly relevant, that is, there is a strong correlation between the instrument (distance) and the endogenous variable (grandparental care).

**TABLE 1:** Effects of grandparental care on parental health and well‐being.

	Grandparental care		F‐statistic	Sample mean	Obs.
Outcomes	OLS	IV			
Mother's health	0.027	−0.138	456.458	3.627	9025
	(0.027)	(0.121)			
Mother's satisfaction with:					
Life	0.018	−0.040	456.712	7.752	9024
	(0.042)	(0.196)			
Education, career	0.187***	0.477*	453.623	7.179	8875
	(0.059)	(0.273)			
Leisure	0.021	0.684**	456.622	6.344	9024
	(0.061)	(0.274)			
Relationship	0.048	−0.110	437.976	7.596	8338
	(0.061)	(0.275)			
Work‐life balance	−0.274***	−0.071	237.039	6.428	3293
	(0.099)	(0.368)			
Child care situation	0.069	0.736*	146.479	8.414	11,412
	(0.073)	(0.437)			
Father's health	0.014	−0.054	281.177	3.796	6217
	(0.031)	(0.139)			
Father's satisfaction with:					
Life	0.025	0.162	280.507	7.785	6215
	(0.047)	(0.216)			
Education, career	0.067	−0.454*	291.429	7.454	6208
	(0.057)	(0.264)			
Leisure	−0.041	−0.174	282.508	6.468	6216
	(0.066)	(0.303)			
Relationship	−0.027	−0.260	281.892	7.695	6212
	(0.072)	(0.344)			
Work‐life balance	−0.084	−0.422	179.398	5.899	3104
	(0.103)	(0.403)			
Child care situation	0.234***	1.567***	81.138	8.415	7399
	(0.087)	(0.501)			

*Note:* Robust standard errors in parentheses. For the outcome “Child care”, robust standard errors clustered at the household level. The outcome variables are all ordinal variables on a scale from 0 (very dissatisfied) to 10 (very satisfied). General: general life satisfaction, Education, career: satisfaction with education and career, Leisure: satisfaction with leisure and hobbies, Relationship: satisfaction with the relationship with the current partner, Work‐life balance: satisfaction with the proportion of time that individuals spend on the job or for vocational training or university education relative to the time that individuals spend on personal life, Child care: satisfaction with the child care situation (on child level, all other outcomes on parental level). The regressions include the control variables listed in Table A1 column (b) for the outcome “Child care” and (c) for all other outcomes.

*Source:* Pairfam (2010–2020), weighted, own calculation.

^*^ *p* < 0.10.

^**^ *p* < 0.05.

^***^ *p* < 0.01.

**TABLE 2:** Results: Child health.

	Grandparental care				
	OLS	IV	F‐statistic	Sample mean	Obs.
Child health					
0–2.9 years	0.026	−0.404	66.900	4.450	1904
	(0.047)	(0.255)			
3–5.5 years	0.030	−0.180	111.015	4.414	3257
	(0.036)	(0.166)			
5.6–10 years	−0.025	−0.385^***^	170.219	4.406	7093
	(0.032)	(0.135)			
0–10 years	0.000	−0.343^***^	199.120	4.406	12,254
	(0.026)	(0.127)			

*Note:* Robust standard errors clustered at the household level in parentheses. The general health variable is an ordinal variable on a scale from 1 (bad health) to 5 (good health). The regressions include the control variables listed in Table A1 column (a).

*Source:* Pairfam (2010–2020), weighted, own calculation.

*p < 0.10.

**p < 0.05.

^***^ *p* < 0.01.

#### 5.1.2 Parental Health and Well‐Being

The effects of grandparental care on parental health and satisfaction are presented in Table 1. The findings for mothers are summarized in the upper panel, while those for fathers are presented in the lower panel. Grandparental care has a positive and statistically significant impact on three satisfaction domains of mothers: education and career, leisure, and the child care situation. Specifically, it leads to a 7% increase in satisfaction with education/career, an 11% increase in satisfaction with leisure, and a 9% increase in satisfaction with the child care situation, when compared to the mean.^22^ In terms of magnitude, the effect sizes are comparable to the impact of an increase in daycare availability for children 3 years and older on maternal life satisfaction (Schmitz 2019). The insignificant coefficients for the other satisfaction and health variables should not be interpreted as null effects since the standard errors are sizable. Therefore, we cannot infer the direction of the effects.

The absence of a significant effect on life satisfaction can be attributed to the predictive ability of specific satisfaction outcomes in different areas for overall life satisfaction. Table A5 illustrates the correlations between life satisfaction and all the satisfaction domains examined in our study. It is clear that satisfaction with the partner relationship carries the highest explanatory weight, while satisfaction with the child care situation is of least importance. As the coefficient for the relationship and work‐life balance satisfaction effect is negative (though not statistically significant), the positive effects in the three other domains are not strong enough to outweigh the importance of relationship satisfaction, ultimately resulting in an insignificant impact on life satisfaction.

A comparison of the IV and OLS estimates reveals that the OLS estimator underestimates the impact of grandparental care on satisfaction with education/career, leisure, and child care. One possible explanation for this is that parents with generally low well‐being are more likely to seek assistance and therefore rely more heavily on grandparental care.

Next, we analyze the impact of grandparental care on paternal health and satisfaction, using the same variables. Similar to mothers, grandparental care has a statistically significant and positive effect on fathers' satisfaction with the child care situation. However, this effect is much larger in magnitude. Specifically, there is an increase of approximately 19% compared to the average. On the other hand, when grandparents provide child care, fathers' satisfaction with their career and education decreases by 6% compared to the average. There is no significant impact on the remaining measures of health and well‐being. It is worth noting that while there is a strong positive effect on satisfaction with child care, this effect does not translate into overall life satisfaction. Satisfaction with education and career has more explanatory power than satisfaction with child care, as shown in Table A5.

#### 5.1.3 Child Health

Table 2 presents the effects on children's health, specifically focusing on general health across three distinct age groups. It is important to remember that the alternative to grandparental care differs depending on the age group. For children under the age of three, the alternative is typically sole parental care. However, this changes for older children. For them, the alternative is either half‐day daycare or school in combination with sole parental care in the afternoon, or all‐day daycare and school combined with parental care.

The reported coefficients indicate that grandparental care has a negative effect on the health of children below the age of 11. Specifically, we find that grandparental care leads to an 8% increase in children's health problems compared to the sample mean. This effect appears to be primarily driven by children of elementary school age, as the coefficient for this subgroup estimation is similarly significant and of similar magnitude to the coefficient for all children combined. However, for children in other age groups, the coefficient is not statistically significant. The decline in health is sizable, when considering that daycare attendance—on average a more intensive care mode—leads to similarly sized declines in health (Baker et al. 2008; Barschkett 2022).

Table 2 also allows for a comparison between the OLS and IV estimates. We note that the OLS estimate (column 1) consistently underestimates the impact of grandparental care on health across all age groups. The OLS estimates suggest that there is no effect on the health of children who receive grandparental care. This finding supports our hypothesis that parents of children with poor health are less likely to seek assistance from grandparents.

### 5.2 Mechanisms

In the following section we provide suggestive evidence for different potential mechanisms which might drive the effects of grandparental care on family health and well‐being. To this end, we conduct various subsample analyses to investigate treatment heterogeneity and examine the relationship between the use of formal and grandparental care.

First, in Table A6, we examine the relationship between attending (all‐day) daycare or school and receiving care from grandparents.^23^ This analysis shows that children who are cared for by their grandparents are less likely to attend all‐day daycare or school. This negative relationship aligns with Figure 2, suggesting that in the afternoon, children are typically cared for by only two caregivers: either parents and grandparents or parents and daycare/school. Additionally, there is a shortage of (all‐day) daycare and all‐day school slots (Gambaro et al. 2024), indicating that grandparents fill the gap in formal care in the afternoon for children 3 years and older and in the morning for children below the age of three. If children who receive care from their grandparents in the afternoon are less likely to attend afternoon programs at school, the negative effect of grandparental care on the health of elementary school‐aged children may be attributed to the different activities organized by grandparents compared to schools. While all‐day schools include homework supervision, sports, arts, music, and playtime in their afternoon curriculum (e.g., Ministry of education of North Rhine Westphalia 2024), less is known about the activities carried out by grandparents and their grandchildren. If grandparents focus less on sports (and other outdoor) activities than schools do, this might explain why grandparental care has negative effects on child health. Additionally, around 10% of children aged three and older are cared for by parents, grandparents, and daycare/school in the afternoon. Bratsch‐Hines et al. (2015) demonstrate that social competencies of children are lower when they experience greater instability of caregivers across childcare settings. Consequently, if children interact with multiple caregivers in different care environments within a single afternoon, this may be too stressful for some, potentially leading to poorer (mental) health outcomes.

To further discuss possible mechanisms, we now proceed with subsample analyses based on child age, parental education, gender of the child, and grandparental age, providing suggestive evidence for different mechanisms. As we only interpret the estimates in the main specification that are statistically significant, we conduct subsample analyses only for these outcomes. We begin by analyzing child health within two specific subgroups. As established in the literature (e.g., Conti et al. 2016), there are known differences in child outcomes based on the child's gender. Therefore, we estimate separate models for boys and girls. Our findings indicate that the negative health effects are primarily associated with boys, as the coefficient for boys is larger in magnitude and statistically more significant (see Table A9). Next, we divide the sample based on the median grandparental age, which is approximately 64 years Table A9 demonstrates that health issues resulting from grandparental care are most noticeable among children who are cared for by grandparents below the median age. This might be due to the fact that grandparents below the median age are more likely to be actively employed, whereas older grandparents are more likely to be retired. As retirement frees up the time resources of grandparents and facilitates the provision of child care (e.g., Tanskanen et al. 2021), retired grandparents may have more time for outdoor activities, cooking healthy meals, and experience less stress, all of which could lead to a higher quality of care.

Additionally, we estimate the effects of grandparental care on parents for different child age groups. The results are presented in Tables A7 and A8. Grandparental care has a positive effect on mothers' satisfaction with education and career, particularly when they have very young children. Moreover, the effect on satisfaction with leisure is mostly observed among mothers with children of elementary school age (5.5–10 years). This finding could possibly be explained by the limited availability of all‐day school slots and the opportunity for mothers to schedule their leisure time in the afternoon with the assistance of grandparental care—as also seen in Table A6. The effect on maternal satisfaction with child care is mostly driven by mothers with children below school age, while for fathers, the estimates for satisfaction with the child care situation are at least significant on the 5% significance level across all age groups and especially large in magnitude for fathers with children below the age of 3. By contrast, we cannot conclude which child age group drives the results for satisfaction with education and career.

Differentiating by parental educational degrees reveals that the positive effect on mothers' satisfaction with education/career and child care is more pronounced for mothers with a university degree compared to those without (Table A7). This may be because highly educated mothers tend to work longer hours and consequently face more challenges in balancing child care and work responsibilities without help from grandparents. Hence, it appears that grandparents are more likely to provide support to highly educated mothers in reconciling child care and work commitments. This finding is in line with other research which shows that the availability of grandparental care leads to an increase in mother's labor supply (Aparicio Fenoll 2020; Bratti et al. 2018; Compton and Pollak 2014; Kanji 2018). As for fathers, the situation is similar: the positive effect on satisfaction with child care is mainly observed among fathers who have a university degree (Table A8).

### 5.3 Validity of the Instrument and Robustness

In this section, we provide several robustness checks to further corroborate our findings and test the validity of the instrument. The results of the robustness checks are depicted in Table A11 for mothers, Table A12 for fathers, and Table A13 for children and show that our findings are generally stable across specifications, although sometimes less statistically significant. Effects on mothers' satisfaction with leisure and child care, fathers' satisfaction with child care, and children's health turn out to be robust across specifications, while the effects on parents' satisfaction with education and career are not as robust.

First, it can be argued that demand for child care increases the likelihood of families living closer to the grandparents (e.g., Chen and Zhang 2018). To test this hypothesis, we investigate whether the distance between parents and grandparents decreases around the time of a child's birth, indicating that either parents moved closer to the grandparents or grandparents moved closer to the parents. The reason for a systematic moving behavior could be to facilitate grandparental child care, which would make distance an endogenous variable. However, our investigation of moving behavior in the year before and after the birth of the firstborn or any child shows no systematic movement toward the grandparents (see Table A10). We further restrict the sample to households that did not move during the observation period. This ensures that we exclude any households that may have moved closer to the grandparents specifically to facilitate child care. The coefficients in our analysis remain stable and significant, with the exception of satisfaction with education and career.

As households living close to the grandparents and those living further away seem to differ in some of their characteristics (see Table A14), we combine our IV estimation with entropy balancing (Hainmueller 2012), a matching strategy that balances controls more effectively than propensity score methods. First, we conduct this matching step and then we run our regular IV estimations. The main idea of entropy balancing is to assign a weight to observations in the “control group” (families living further than 30 min away), causing the “control group's” distributions of the selected covariates to match those of the “treatment group” (families living closer than 30 min) on the mean. Consequently, our set of covariates has the same means in both groups. These weights are then applied to our IV estimations. The effects remain very similar; we still depict highly significant effects on children's health, parental satisfaction with child care, and maternal satisfaction with leisure. However, the negative effect on paternal satisfaction with education and career is no longer statistically significant.

We provide additional evidence that our specification isolates the impact of grandparental care on parental well‐being by utilizing a sample of childless households. Specifically, we directly regress our outcomes on the distance to the “grandparents”^24^ which should not exhibit an effect if grandparental care is the only important channel. The results reveal that the point estimates are negligible in magnitude and that there are no statistically significant effects of distance on well‐being for both childless women and men.^25^

Next, we only consider the distance to an individual's parents‐in‐law (rather than the distance to any grandparent) as an instrument when estimating the effects of grandparental care on parental outcomes. The rationale behind this is that the relationship extends beyond child care and is typically closer to one's own parents than to one's parents‐in‐law (e.g., Del Boca et al. 2018). Therefore, if the distance to one's own parents has any effect on parental satisfaction through factors other than child care that we cannot account for, this should be eliminated when using the distance to the parents‐in‐law. In general, the results are similar to our main findings, but they are less statistically significant due to substantially smaller sample sizes. The effects on parental satisfaction with their career are smaller and no longer statistically significant.

Although we have demonstrated that grandparental care appears to be the primary channel through which distance affects child and parental outcomes, we further test the robustness of our results by relaxing the exclusion restriction. Following the approach of Conley et al. (2012), we provide bounds on the second‐stage effect of grandparental care on parental and child outcomes, allowing for a degree of endogeneity in the instrument. We begin by estimating the reduced form effect of the instrument (Tables A11, A12, and A13, column 2 of “Relaxing exclusion restriction”). Subsequently, we calculate the bounds for the second‐stage effects (Tables A11, A12, and A13, columns 3 and 4 of “Relaxing exclusion restriction”), permitting the direct effect of the instrument on our outcomes (γ) to range from zero (perfectly exogenous) up to 30% of the reduced form effect.^26^ The bounds for the second‐stage estimate exclude zero as long as the direct effect of the instrument $\left(\gamma_{\max}\right)$ is less than 30% of the reduced form effect. However, child health is only robust to allowing the direct effect to be at most 21% of the reduced form effect, while parental satisfaction with education and career is less robust to endogeneity. We conclude that most of our effects remain robust even under a substantial degree of instrument endogeneity.

To account for the fact that the length (in years) of exposure to grandparental care may affect the results, we construct a grandparental care variable that averages past (over the past 3 years) and current grandparental care.^27^ The findings closely align with our baseline estimates for most outcomes.

Additional robustness checks, such as employing a placebo outcome, excluding households living further than 3 h away but using regular grandparental care, using different definitions of the instrument, and including or excluding additional control variables or imputing missing values, further support the robustness of our results. Correcting for multiple hypothesis testing yields a *p*‐value of 0.077 for maternal satisfaction with leisure while parental satisfaction with education and career is no longer significant. This demonstrates that our results for maternal satisfaction with leisure hold up even when accounting for the number of hypotheses tested.^28^ Overall, the results regarding parental satisfaction with child care, maternal satisfaction with leisure, and child health are the most robust, while the results regarding parental satisfaction with career should be interpreted with caution.

## 6 Conclusion

Our analysis contributes to the existing literature on the determinants of health in the family context and the intergenerational effects of regular grandparental care on the outcomes of parents and children. Our results are particularly interesting because grandparental care continues to play an important role in the “care puzzle” for many families, despite efforts to expand formal care options. Furthermore, we extend the literature on the effects of grandparental care by estimating the causal effects on family health and well‐being, as measured by the subjective health and well‐being of mothers, fathers, and children. To overcome endogeneity between grandparental care and our outcomes, we employ an instrumental variable approach, using the distance to the grandparents as our instrument, which we combine with entropy balancing. We also provide various robustness checks to support the validity of our instrument.

Using a representative German panel data set, our results provide evidence of mainly positive effects on various aspects of parental satisfaction, but negative effects on children's subjective health. Specifically, we show that grandparental care leads to improvements in parental satisfaction with child care and maternal satisfaction with leisure. Therefore, grandparenting is beneficial for the parent's generation, particularly for mothers. This is plausible because, on average, mothers are still the primary caretakers and thus benefit the most. Through this effect, it may also have long‐term benefits for the child's development, as maternal well‐being has been found to positively impact child outcomes (see Datta Gupta et al. 2023). In the short term, however, grandparental care seems to have adverse effects on children's health.

The positive effects of grandparental care on parents' satisfaction with child care and mothers' satisfaction with leisure are highly robust, regardless of the various specifications, sample restrictions, and instruments used. However, the effects observed for parental satisfaction with their education and career are less stable and should therefore be interpreted with caution. When comparing our effects to the impact of daycare attendance on maternal life satisfaction, as illustrated for instance by Schmitz (2019), it becomes evident that our effects, ranging from 9 to 11%, are of similar magnitude.

Additionally, we provide evidence that grandparental care has a negative impact on the health of elementary school children (8%). Studies on the health effects of other forms of care, such as daycare, yield mixed results. For instance, in their study, Cornelissen et al. (2018) identify positive health effects of daycare that are of larger magnitude compared to our findings. Specifically, they report a 25% reduction in the need for “compensatory sports” upon entering school. On the other hand, Baker et al. (2008) uncover adverse health effects resulting from a major expansion of daycare in Quebec, Canada, amounting to 9% compared to the average. Similarly, Barschkett (2022) demonstrates that daycare attendance results in short‐term increases in infectious disease prevalence, followed by comparable long‐term decreases, with effects similar in magnitude (five to six percent) to those observed for grandparental care. Since this is the first piece of causal evidence concerning the impact of grandparental care on overall child health, it is not possible to make direct comparisons with other estimates on this care mode.

Overall, our results indicate that in addition to parental care and daycare, regular child care provided by informal caregivers such as grandparents also has an impact on parents, children, and the family as a whole. In addition to our analysis, to fully understand the underlying mechanisms behind these effects, a more detailed analysis is needed. To investigate further, for instance, data that includes the activities grandparents engage in with their grandchildren would be necessary (Sadruddin et al. 2019). Additionally, like other forms of care, more information on the quality of the time spent in child care is required (Milovanska‐Farrington 2021). Lastly, it is important to examine the long‐term effects to determine whether the positive effects on maternal satisfaction improve child outcomes and other maternal factors, thereby demonstrating additional indirect effects of grandparental care. The method employed in this paper is an instrumental variable approach. Since this method only reveals effects for compliers, and we demonstrate that in our setting compliers differ from always‐takers and never‐takers in specific characteristics, caution is necessary when generalizing the results to other populations. A key advantage of studying the research question within the German context is that the development of formal child care mirrors trends in many other countries, while the use of informal child care aligns with the European average. This similarity facilitates the extension of our findings to other European countries.

Our paper provides evidence that family health and well‐being can be affected by interfamilial interactions. Hence, policymakers should not only focus on daycare, but also on informal care when aiming to improve societal health. Considering the positive effects of grandparental care on parental well‐being, discussions could be held on implementing national insurance credits for grandparents who care for dependent children, which would contribute to their retirement income, similar to what has been done in the UK. Additionally, introducing grandparental leave and benefits, as seen in Sweden (Olsen 2024), could be measures to support grandparental care. However, considering the mixed findings regarding the effects of grandparental care on grandparents' health and well‐being (Danielsbacka et al. 2019; Eibich and Zai 2024), it is crucial to also account for the grandparents' perspective before implementing policies that promote grandparental care. Considering the negative effects of grandparental care on some children's health, our results might also suggest that too many care modes in one day could have negative effects on children. To address this, politicians could consider policies that support longer daycare hours, shorter working days, or other measures to reduce the “child penalty” that employed parents may face when the opening hours of daycare centers do not align with their working schedules (e.g., Jessen 2022).

## Conflicts of Interest

The authors declare no conflicts of interest.

## Data Availability

The data that support the findings of this study are available from FDZ pairfam. Restrictions apply to the availability of these data, which were used under license for this study. Data are available from https://www.pairfam.de/en/data/data‐access/ with the permission of FDZ pairfam.

## Endnotes

^1^
The term “daycare” refers to all forms of formal child care provided by professionals outside the family. The term “parental child care” refers to all child care provided by the child's mother or father. “Grandparental care” describes the situation in which grandparents care for their grandchildren on a regular basis, that is, excluding emergency care.

^2^
For a literature overview, primarily focusing on studies that display non‐causal associations between grandparental care and parental and child health, see Hank et al. (2018).

^3^
Health and well‐being are two closely related concepts that are difficult to distinguish from each other. The WHO defines “Health is a state of complete physical, mental and social well‐being and not merely the absence of disease or infirmity” (WHO 1948). Therefore, in this study we use the terms health and well‐being as measures of physical and mental health as well as overall and domain‐specific life satisfaction. Such subjective evaluations are considered valid, reliable and cost‐effective means of health assessment (Kaplan and Baron‐Epel 2003).

^4^
Eibich and Zai (2024) show that grandparental care has adverse effects for grandparents' self‐reported health, limitations in daily activities and depressive symptoms. These health challenges may result in grandparents being more likely to cancel their caregiving commitments unexpectedly, potentially disrupting child care arrangements.

^5^
Caring for grandchildren positively influences grandmother's verbal fluency (e.g., Arpino and Bordone 2014). However, it also reduces grandparents' participation in social activities (e.g., Arpino and Bordone 2017) and grandmother's labor supply (e.g., Backhaus and Barslund 2021; Frimmel et al. 2020). Finally, Danielsbacka et al. (2019) find a decrease in grandparents' reported limitations with activities of daily living but no change in reported overall health, life satisfaction or depressive symptoms. By contrast, Eibich and Zai (2024) demonstrate that grandparental care adversely affects grandparents' physical functioning, subjective health and depressive symptoms, particularly among grandmothers.

^6^
Sadruddin et al. (2019) provide a comprehensive overview of non‐causal evidence on the relationship between grandparental care and child health.

^7^
These findings align with Zanasi et al. (2023), demonstrating that over 23 percent of grandparents provided weekly childcare in Germany in 2020.

^8^
In comparison, the average female employment rate in OECD countries was 72.3 percent in 2021 (e.g., OECD 2023b).

^9^
Except for the satisfaction with child care, as this variable is collected at the child level.

^10^
This approximation is valid because in 97 percent of the households in our sample, either none or all of the children are cared for by the grandparents.

^11^
Only 0.02 percent of children under the age of 11 have no living grandparents.

^12^
The six categories are: “we live in one house”, “less than 10 min”, “10 min to less than 30 min”, “30 min to less than 1 h”, “1 h to less than 3 h” and “3 h or more”.

^13^
In robustness checks, we test whether our results are sensitive to two different definitions of the instrument (e.g., using an ordinal instrument and 1 hour as the cutoff). See Appendix E.

^14^
Studies based on other data sources corroborate these findings for Germany and other European countries. For example, Isengard (2013), analyzing SHARE data, reports that 62% of adult children reside within 25 km of their parents, which closely aligns with the European average of 68%.

^15^
It appears that some (8%) of those households in our sample, who live further than 3 hours away from all grandparents, still report using grandparental care regularly. We exclude these households in a robustness check, and it does not affect our results. The results are available upon request.

^16^
Several control variables in our dataset contain missing values, attributable to either non‐response or the inconsistent inclusion of certain questions across survey waves. We impute time‐invariant control variables (e.g., migration background) with values from previous waves. To address potential bias from systematic non‐response patterns, we employ mean imputation for continuous variables and create an additional category for missing values in discrete and binary variables to include observations with non‐responses in a robustness check.

^17^
For 2020, we only include households surveyed before March 15, prior to the onset of the COVID‐19 pandemic in Germany.

^18^
All outcomes are measured using ordinal variables. Ferrer‐i Carbonell and Frijters (2004) demonstrate that life satisfaction can be treated as cardinal, offering the advantage of easier interpretation compared to methods that treat variables as strictly ordinal. Following established practices in the literature, we treat these variables as cardinal in our analysis.

^19^
This is because the observations of different children in one household might be correlated with each other, and, as a result, the i.i.d. assumption would not hold. Robust standard errors are used for all other parental outcomes.

^20^
Theoretically, the population in an IV approach consists of four groups, the fourth being defiers. Defiers are individuals who would only use grandparental care if they lived far away from the grandparents, but this scenario is unlikely in our context. As is common in the literature, we assume that there are no defiers in our sample.

^21^
We tested three additional potential instruments using a pension reform in Germany, the parents' birth order, and the gender of the oldest sibling of both parents. All three instruments proved to be weak (small first stage F‐statistic).

^22^
The percent changes are calculated by relating the coefficients to the corresponding sample means, that is, $\frac{\beta_{IV}+Mean}{Mean}$.

^23^
Similar to Figure 1, the different care settings are not mutually exclusive; for example, a child may attend all‐day daycare or school while also receiving care from their grandparents.

^24^
Referring to the parents or parents‐in‐law of childless adults, who represent our grandparent generation.

^25^
Because individuals in childless households are generally younger than parents in households with children in *pairfam*, we exclude the youngest quartile of the sample in additional regressions to ensure comparability between the childless sample and our main sample. Even in these analyses, we still do not find any effects of distance on well‐being.

^26^
We implement this using Stata's *plausexog* command, employing 30% of the reduced form effect as $\gamma_{\max}$ (Clarke and Matta 2018).

^27^
In this regression, we use the average distance between grandparents and the family over the past 3 years as the instrument.

^28^
To implement the correction for multiple hypothesis testing, we apply the Romano Wolf procedure (Clarke and Matta 2018) using the Stata command *rwolf*. As *rwolf* can only be conducted within one data set, we ran the test only for parental satisfaction outcomes (excluding satisfaction with child care as it is part of another subsample of the data set), separately for mothers and fathers.

## Appendix B Further Information on the Data

### Pairfam

*Pairfam* respondents are equally distributed among the birth cohorts 1991–1993, 1981–1983, and 1971–1973 and the first wave of the sample consisted of 12,400 respondents (Huinink et al. 2011). These individuals are called “anchor persons.” Approximately one half of the anchors are male, and the other half are female. In addition, if anchors and anchors' partners agreed, partners were surveyed from the first wave onwards. The response rate for partners lies at about 52%.^29^ *Pairfam* is a multi‐actor survey. In addition to anchors and partners, children (aged 8–15 years) and parents of anchors are surveyed separately. Furthermore, anchors and partners are questioned about their children (biological, adopted, foster, and stepchildren of anchors living in one household) and parents in their own questionnaires in detail (Huinink et al. 2011). This detailed information on three generations makes *pairfam* particularly suitable for our analysis. Since the child survey only includes children above the age of 7 and the parent survey suffers from a low response rate, we focus on the information obtained from the anchor and partner questionnaires in our analysis.

### Summary Statistics

Table A2 includes summary statistics of selected control variables (based on the sample on child level), showing mean and standard deviation across all observations. It can be seen that 23% of children in the sample are cared for by the grandparents on a regular basis^30^ and almost 70% of families live close to at least one grandparent.

The sample is, on average, highly educated, as in almost half the families at least one partner holds a university degree. Generally, *pairfam* includes a slightly more highly educated sample than the German population (Wetzel et al. 2021). In terms of migration background, 12% of children have at least one parent who was born outside Germany. Half the children in the sample are girls and children are on average 5 years old. Furthermore, 91% of parents are cohabiting.

**TABLE A1:** Control variables.

Variable	Definition	Type	To estimate effects on		
			Children's	Parents'	
			(a)	(b)	(c)
Parental variables					
Age	Mother's age	Cont	✓		
	Individual age	Cont		✓	✓
Post‐secondary education	Highest degree in household, 1–3	Ord	✓		
	Individual education, 3 levels	Ord		✓	✓
Religion	One parent religious	Bin	✓		
	Individual religion, 1–7	Cat		✓	✓
Migration background	One parent has direct background	Bin		✓	
	Individual has direct background	Bin		✓	✓
Partner information	Partner answered questionnaire	Bin	✓		
Parental goals	Importance nutrition and exercise, 1–10	Ord	✓		
Pregnancy	Mother is pregnant	Bin	✓	✓	✓
Cohabitation	Parents live together	Bin	✓	✓	✓
Widowhood	One parent is widowed	Bin	✓	✓	
	Individual is widowed	Bin			✓
Only child	At least one parent is only child	Bin	✓		
	Individual is only child	Bin		✓	✓
Child variables					
Sex	Child's sex	Bin	✓	✓	
	Children in HH: male, female, mixed	Cat			✓
Child age	In months	Cont	✓	✓	
	Age of youngest child in months	Cont			✓
Number children in HH	Total	Cont	✓	✓	
	Nr. children 0–2 years	Cont			✓
	Nr. children 3–5 years	Cont			✓
	Nr. children 6–10 years	Cont			✓
	Nr. other children	Cont			✓
Birth order	Age in comparison to sibling's age	Ord	✓	✓	
Daycare use	Child (0–5 years) in daycare	Bin	✓	✓	
	Number of children (0–5 years) in daycare	Cont			✓
Grandparent variables					
School education	Anchor's mother, 1–3	Ord	✓	✓	✓
	Anchor's father, 1–3	Ord	✓	✓	✓
Age	Mean of all living grandparents	Cont	✓	✓	✓
Household (HH) variables					
Household income	Logarithmic, in 1000€	Cont	✓	✓	✓
Year	Number according to wave number	Cat	✓	✓	✓
Federal state	1–16	Cat	✓	✓	✓
Community size	1–7	Ord	✓	✓	✓

*Note:* This table shows which variables are used to estimate the effect of grandparental care on: (a) Child's health; (b) Parental satisfaction with childcare; (c) Other parental satisfaction outcomes. Types: Bin (binary), Cat (categorical), Cont (continuous), Ord (Ordinal).

*Source:* Pairfam, 2009–2019.

**TABLE A2:** Summary statistics.

	Percentage/mean (SD)
Grandparent care	23.32%
Grandparents live 30 min or closer	68.85%
Mother's labor force status (in percent)	
Mother not working	36.33%
Mother working part‐time	42.67%
Mother working full‐time	18.88%
Household's highest parental school degree (in percent)	
No/lower secondary degree	5.92%
Upper secondary/vocational degree	45.51%
University degree	48.57%
One parent has migration background	11.90%
Household net income (in euro)	3416.56 (2430.79)
Age mother (in years)	34.02 (7.90)
Sex child: Male	50.88%
Number of children in household	2.04 (0.99)
Age child (in years)	4.90 (3.10)
Cohabitation with partner	91.07%
Observations	29,177

*Note:* Conditional on non‐missing sample.

*Source:* Pairfam 2010–2020, weighted, own calculations.

**TABLE A3:** Profiling compliers.

Variable	Always‐taker	Never‐taker	Complier
Proportion	0.081	0.694	0.225
Mother not working	0.220 (0.015)	0.404 (0.004)	0.241 (0.010)
Mother working part‐time	0.447 (0.018)	0.392 (0.004)	0.481 (0.010)
Mother working full‐time	0.281 (0.015)	0.187 (0.003)	0.273 (0.009)
No/lower secondary degree	0.040 (0.007)	0.050 (0.002)	0.016 (0.004)
Upper secondary/vocational degree	0.312 (0.017)	0.509 (0.004)	0.225 (0.011)
University degree	0.647 (0.017)	0.442 (0.004)	0.760 (0.011)
One parent has migration background (share)	0.094 (0.010)	0.071 (0.002)	0.124 (0.006)
Household net income (in euro)	3723.667 (82.227)	3275.983 (18.304)	3912.180 (49.352)
Age mother (in years)	33.201 (0.375)	33.982 (0.055)	36.277 (0.172)
Number of children in household	1.767 (0.031)	2.151 (0.008)	2.075 (0.020)
Age child (in years)	4.691 (0.103)	4.971 (0.027)	5.073 (0.065)
Cohabitation with partner (share)	0.831 (0.014)	0.911 (0.002)	0.931 (0.007)
Grandparents' age	63.637 (0.298)	62.470 (0.063)	65.238 (0.173)

*Note:* Conditional on non‐missing sample, except grandparental health. Means and standard deviations of the different groups are produced using Stata's command *ivdesc*. SDs in parentheses. *Source:* Pairfam 2010–2020, weighted, own calculations.

## Appendix C First Stage Results

**TABLE A4:** First stage results.

	Sample:		
	Child	Mother	Father
Distance	0.241***	0.239***	0.238***
	(0.017)	(0.013)	(0.016)
Observations	12,254	6196	4489
F‐statistic	198.640	484.553	365.483
Adjusted R‐squared	0.106	0.151	0.154

*Note:* The outcome variable in these regressions is grandparental care and the variable of interest is our instrument, the minimum distance between the family and the grandparents. The different columns show the first stage regression for three different samples: children, mothers, fathers. Robust standard errors in parentheses. Conditional on no missings in the outcome and control variables (see Table A1). *Source:* Pairfam (2010–2020), weighted, own calculation.

**p* < 0.10.

***p* < 0.05.

^***^ *p* < 0.01.

## Appendix D Additional Analyses

**TABLE A5:** Domain‐specific and life satisfaction.

Satisfaction with	Mothers	Fathers
Education, career	0.140***	0.190***
	(0.015)	(0.015)
Leisure	0.148***	0.107***
	(0.014)	(0.014)
Relationship	0.274***	0.240***
	(0.015)	(0.015)
Work‐life balance	0.075***	0.090***
	(0.011)	(0.011)
Child care situation	0.070***	0.064***
	(0.016)	(0.014)
Obs.	5628	5947

*Note:* The table shows results of regressions of mother's and father’ life satisfaction on variables capturing domain specific satisfaction. Robust standard errors in parentheses.

*Source:* Pairfam (2010–2020), weighted, own calculation.

**p* < 0.10.

***p* < 0.05..

^***^ *p* < 0.01.

**TABLE A6:** Relationship between formal and informal care usage.

	OLS	Obs.
0–2 years in daycare	−0.075^**^	2884
	(0.022)	
3–5.5 years in allday daycare	−0.100^***^	4201
	(0.021)	
5.5–10 years in allday school	−0.068^**^	6826
	(0.020)	
3–10 years in allday daycare/school	−0.075^**^	12,439
	(0.022)	

*Note:* OLS regression with daycare/allday care or school as dependent variable and grandparental care as independent variable. The regressions include the control variables listed in Table A1 column (a). Robust standard errors in parentheses.

*Source:* Pairfam (2010–2020), weighted, own calculation.

**p* < 0.10.

^**^ *p* < 0.05.

^***^ *p* < 0.01.

**TABLE A7:** Subsample Analysis: Mothers' satisfaction.

	Grandparental care	F‐statistic	Sample mean	Obs.
Satisfaction with education, career				
Children's age				
0–2.9 years	1.108** (0.509)	133.319	7.130	3059
3–5.5 years	0.223 (0.421)	166.761	7.227	3442
5.6–10 years	0.644* (0.375)	258.128	7.156	4676
Mother's education				
University degree	0.540* (0.286)	258.383	7.657	3233
No university degree	0.542 (0.480)	186.995	6.947	5642
Satisfaction with leisure				
Children's age				
0–2.9 years	0.760 (0.514)	137.629	6.087	3161
3–5.5 years	0.326 (0.430)	167.983	6.181	3503
5.6–10 years	1.051*** (0.388)	256.239	6.374	4738
Mother's education				
University degree	0.653** (0.323)	260.417	6.378	3295
No university degree	0.791* (0.448)	188.761	6.314	5729
Satisfaction with child care				
Children's age				
0–2.9 years	1.002 (0.969)	45.329	8.550	2015
3–5.5 years	1.055* (0.635)	71.036	8.436	3071
5.6–10 years	0.561 (0.439)	140.636	8.347	6326
Mother's education				
University degree	1.061* (0.544)	71.454	8.445	5995
No university degree	0.810 (0.832)	66.700	8.372	5417

*Note:* Robust standard errors in parentheses. For the outcome satisfaction with “Child care”, robust standard errors clustered at the household level. The outcome variables are all ordinal variables on a scale from 0 (very dissatisfied) to 10 (very satisfied). Leisure: satisfaction with leisure and hobbies, Child care: satisfaction with the child care situation (on child level, all other outcomes on parental level). The regressions include the control variables listed in Table A1 column (b) for the outcome satisfaction with “Child care” and (c) for all other outcomes.

*Source:* Pairfam (2010–2020), weighted, own calculation.

^*^ *p* < 0.10.

^**^ *p* < 0.05.

^***^ *p* < 0.01.

**TABLE A8:** Subsample Analysis: Fathers' satisfaction.

	Grandparental care	F‐statistic	Sample mean	Obs.
Satisfaction with education, career				
Children's age				
0–2.9 years	0.317 (0.350)	162.288	7.473	2545
3–5.5 years	−0.353 (0.292)	239.321	7.435	2550
5.6–10 years	0.308 (0.332)	201.099	7.372	3059
Father's education				
University degree	0.086 (0.202)	369.099	7.734	2858
No university degree	−0.360 (0.452)	120.324	7.174	3327
Satisfaction with child care				
Children's age				
0–2.9 years	2.073** (0.809)	43.094	8.654	1426
3–5.5 years	1.152** (0.465)	82.938	8.371	2071
5.6–10 years	1.651*** (0.504)	65.136	8.327	3902
Father's education				
University degree	1.490*** (0.451)	77.731	8.406	4273
No university degree	1.370 (0.904)	30.941	8.394	3126

*Note:* Robust standard errors in parentheses. For the outcome “Child care”, robust standard errors clustered at the household level. The outcome variables are all ordinal variables on a scale from 0 (very dissatisfied) to 10 (very satisfied). Education/career: Satisfaction with education and career, Child care: satisfaction with the child care situation (on child level, all other outcomes on parental level). The regressions include the control variables listed in Table A1 column (b) for the outcome “Child care” and (c) for all other outcomes.

*Source:* Pairfam (2010–2020), weighted, own calculation.

^*^ *p* < 0.10.

^**^ *p* < 0.05.

^***^ *p* < 0.01.

**TABLE A9:** Subsample Analysis: Child health.

	Grandparental care	F‐statistic	Sample mean	Obs.
Child's gender				
Boys	−0.428^**^ (0.180)	126.285	1.622	6614
Girls	−0.231 (0.143)	145.452	1.540	6385
Grandparents' health				
GP age above median	−0.305^**^ (0.147)	138.292	1.550	7257
GP age below/equal median	−0.459^**^ (0.204)	84.372	1.622	5742

*Note:* Robust standard errors clustered at the household level in parentheses. The general health variable is an ordinal variable on a scale from 1 (bad health) to 5 (good health). The regressions include the control variables listed in Table A1 column (a).

*Source:* Pairfam (2010–2020), weighted, own calculation.

Abbreviation: GP = grandparent.

**p* < 0.10.

^**^ *p* < 0.05.

****p* < 0.01.

## Appendix E Robustness Checks

**TABLE A10:** (Grand‐)parental moving behavior before and after the birth of a child.

	General movement	Move toward	Move away from
In the year before child birth			
Considering any grandparents	0.0037	0.0032	−0.0098
	(0.019)	(0.017)	(0.016)
*Observations*	22,251	22,251	22,251
Considering mother's parents	0.0182	0.0193	−0.0034
	(0.017)	(0.013)	(0.012)
*Observations*	22,250	22,250	22,250
Considering father's parents	−0.0126	−0.0162	0.0004
	(0.016)	(0.013)	(0.012)
*Observations*	20,904	20,904	20,904
In the year after child birth			
Considering any grandparents	0.0033	0.0154	−0.0038
	(0.015)	(0.013)	(0.013)
*Observations*	22,251	22,251	22,251
Considering mother's parents	0.0220	0.0099	0.0114
	(0.013)	(0.011)	(0.010)
*Observations*	22,250	22,250	22,250
Considering father's parents	−0.0104	0.0057	−0.0136
	(0.013)	(0.011)	(0.010)
*Observations*	20,904	20,904	20,904

*Note:* Estimated using OLS. Standard errors in parentheses. All regressions include individual and household controls described in Table A1 column (a). The sample size differs to that in the main specification as the analysis includes also parents before child birth.

*Source:* Pairfam (2009–2020), own calculations.

**p* < 0.10.

***p* < 0.05.

****p* < 0.01.

**TABLE A11:** Robustness: Mothers' satisfaction.

	Grandparental care	F‐statistic	Sample mean	Obs.
Satisfaction with education, career				
Main estimate	0.477* (0.273)	453.623	7.179	8875
Exclusion of movers	0.308 (0.277)	473.168	7.255	6894
Entropy balancing	0.743^**^ (0.343)	348.973	7.089	8875
Childless households	0.145 (0.131)		7.337	1132
Distance to parents‐in‐law	0.149 (0.372)	193.423	7.272	4730
Average current and past GPC	0.550^*^ (0.305)	633.553	7.199	5545
Exclusion of HH far away using GPC	0.477^*^ (0.273)	453.623	7.179	8875
Discrete instrument	0.234 (0.227)	652.290	7.179	8875
Binary instrument (< 1h vs. ≥ 1h)	0.079 (0.323)	376.465	7.179	8875
Including emot. closeness	0.315 (0.279)	433.025	7.177	8866
Including freq. contact	0.228 (0.329)	307.670	7.179	8875
Including pre‐birth satisfaction	1.300^**^ (0.525)	98.061	7.087	2348
Including labor force status	0.371 (0.271)	440.043	7.181	8829
Excluding income	0.559^*^ (0.288)	370.413	7.209	8241
Controls with replaced missings	0.509** (0.202)	952.475	7.047	17,872
	*Reduced form effect*	$\beta_{\mathrm{lowerbound}}$	$\beta_{\mathrm{upperbound}}$	$\gamma_{\max}$
Relaxing exclusion restriction	0.115* (0.066)	−0.212	1.044	0.035
Satisfaction with leisure				
Main estimate	0.684** (0.274)	456.622	6.344	9024
Exclusion of movers	0.417 (0.286)	468.028	6.363	7006
Entropy balancing	0.869** (0.345)	351.303	6.281	9024
Childless households	0.087 (0.151)		7.062	1134
Distance to parents‐in‐law	0.818** (0.392)	189.783	6.461	4805
Average current and past GPC	0.402 (0.313)	640.455	6.312	5654
Exclusion of HH far away using GPC	0.684** (0.274)	456.622	6.324	9024
Discrete instrument	0.536** (0.226)	661.348	6.335	9024
Binary instrument (< 1h vs. ≥ 1h)	0.544^*^ (0.323)	378.558	6.335	9024
Including emot. closeness	0.538^*^ (0.280)	436.855	6.335	9015
Including freq. contact	0.610^*^ (0.335)	311.422	6.335	9024
Including pre‐birth satisfaction	0.606 (0.555)	96.607	6.237	2415
Including labor force status	0.661^**^ (0.277)	445.549	6.338	8978
Excluding income	0.964^***^ (0.295)	374.334	6.358	8405
Controls with replaced missings	0.524** (0.205)	957.031	6.328	18,120
	*Reduced form effect*	$\beta_{\mathrm{lowerbound}}$	$\beta_{\mathrm{upperbound}}$	$\gamma_{\max}$
Relaxing exclusion restriction	0.187*** (0.067)	0.004	1.373	0.056
Satisfaction with childcare				
Main estimate	0.736* (0.437)	146.479	8.414	11,412
Exclusion of movers	0.969^*^ (0.538)	104.940	8.467	8414
Entropy balancing	1.322^**^ (0.590)	142.183	8.322	11,412
Distance to parents‐in‐law	0.756 (0.684)	38.427	8.468	5886
Average current and past GPC	0.735 (0.530)	119.062	8.446	6678
Exclusion of HH far away using GPC	0.736^*^ (0.437)	146.479	8.407	11,412
Discrete instrument	0.725^**^ (0.365)	202.672	8.407	11,412
Binary instrument (< 1h vs. ≥ 1h)	0.805 (0.534)	129.650	8.407	11,412
Including emot. closeness	0.604 (0.436)	139.005	8.406	11,407
Including freq. contact	0.638 (0.536)	97.592	8.407	11,412
Including labor force status	0.747^*^ (0.445)	145.824	8.453	10,058
Excluding income	0.941^**^ (0.448)	130.162	8.426	12,318
Controls with replaced missings	1.014** (0.417)	143.409	8.359	13,131
	*Reduced form effect*	$\beta_{\mathrm{lowerbound}}$	$\beta_{\mathrm{upperbound}}$	$\gamma_{\max}$
Relaxing exclusion restriction	0.155*** (0.053)	0.024	1.229	0.046

*Note:* Robust standard errors in parentheses. For the outcome “Child care”, robust standard errors clustered at the household level. The outcome variables are all ordinal variables on a scale from 0 (very dissatisfied) to 10 (very satisfied). Child care: satisfaction with the child care situation (on child level, all other outcomes on parental level), Leisure: satisfaction with leisure and hobbies. The regressions include the control variables listed in Table A1 column (b) for the outcome “Child care” and (c) for all other outcomes.

*Source:* Pairfam (2010–2020), weighted, own calculation.

^*^ *p* < 0.10.

^**^ *p* < 0.05.

^***^ *p* < 0.01.

**TABLE A12:** Robustness: Fathers' satisfaction.

	Grandparental care	F‐statistic	Sample mean	Obs.
Satisfaction with education and career				
Main estimate	−0.454* (0.264)	291.429	7.454	6208
Exclusion of movers	−0.499^**^ (0.250)	351.779	7.460	5064
Entropy balancing	−0.356 (0.357)	218.880	7.425	6185
Childless households	−0.147 (0.212)		7.715	938
Distance to parents‐in‐law	0.131 (0.256)	256.907	7.411	4404
Average current and past GPC	−0.528^*^ (0.281)	438.966	7.525	4033
Exclusion of HH far away using GPC	−0.462^*^ (0.264)	290.426	7.421	6185
Discrete instrument	−0.047 (0.217)	435.389	7.421	6185
Binary instrument (< 1h vs. ≥ 1h)	−0.723^***^ (0.271)	323.697	7.421	6185
Including emot. closeness	−0.648^**^ (0.277)	264.468	7.421	6183
Including freq. contact	−0.951^***^ (0.347)	181.016	7.421	6185
Including pre‐birth satisfaction	−0.638^*^ (0.377)	95.704	7.646	2079
Including labor force status	−0.512^*^ (0.263)	283.768	7.422	6168
Excluding income	−0.540^**^ (0.267)	309.134	7.413	6520
Controls with replaced missings	0.152 (0.224)	643.003	7.259	14,760
	*Reduced form effect*	$\beta_{\mathrm{lowerbound}}$	$\beta_{\mathrm{upperbound}}$	$\gamma_{\max}$
Relaxing exclusion restriction	−0.134** (0.063)	−1.089	0.121	−0.040
Satisfaction with childcare				
Main estimate	1.567*** (0.501)	81.138	8.415	7399
Exclusion of movers	1.595^***^ (0.526)	82.004	8.438	5702
Entropy balancing (binary instrument)	2.012^***^ (0.495)	84.412	8.269	7399
Distance to parents‐in‐law	1.898^***^ (0.551)	53.900	8.417	5328
Average current and past GPC	1.342^**^ (0.554)	89.261	8.407	4441
Exclusion of HH far away using GPC	1.567^***^ (0.501)	81.138	8.401	7399
Discrete instrument	1.576^***^ (0.415)	103.139	8.401	7399
Binary instrument (< 1h vs. ≥ 1h)	1.719^***^ (0.587)	73.219	8.401	7399
Including emot. closeness	1.411^***^ (0.520)	73.587	8.401	7399
Including freq. contact	1.460^**^ (0.644)	52.007	8.401	7399
Including labor force status	1.480^***^ (0.481)	84.420	8.473	6720
Excluding income	1.575^***^ (0.496)	81.147	8.394	7781
Controls with replaced missings	1.513*** (0.457)	119.702	8.301	13,756
	*Reduced form effect*	$\beta_{\mathrm{lowerbound}}$	$\beta_{\mathrm{upperbound}}$	$\gamma_{\max}$
Relaxing exclusion restriction	0.334*** (0.058)	0.561	2.112	0.100

*Note:* Robust standard errors in parentheses. For the outcome “Child care”, robust standard errors clustered at the household level. The outcome variables are all ordinal variables on a scale from 0 (very dissatisfied) to 10 (very satisfied). Child care: satisfaction with the child care situation (on child level, all other outcomes on parental level), Leisure: satisfaction with leisure and hobbies. The regressions include the control variables listed in Table A1 column (b) for the outcome “Child care” and (c) for all other outcomes.

*Source:* Pairfam (2010–2020), weighted, own calculation.

^*^ *p* < 0.10.

^**^ *p* < 0.05.

^***^ *p* < 0.01.

**TABLE A13:** Robustness: Child health.

IV: Grandparental care		F‐statistic	Sample mean	Obs.
Main estimate	−0.343^***^ (0.127)	199.120	4.406	12,254
Exclusion of movers	−0.236^*^ (0.139)	198.456	4.431	9594
Entropy balancing (binary instrument)	−0.279^**^ (0.121)	278.104	4.418	12,999
Average current and past GPC	−0.293^**^ (0.138)	201.649	4.441	8949
Exclusion of HH far away using GPC	−0.322^**^ (0.128)	210.135	4.418	12,999
Discrete instrument (< 30 min vs. ≥ 30 min)	−0.221^**^ (0.105)	265.742	4.418	12,999
Binary instrument (< 1h vs. ≥ 1h)	−0.245 (0.153)	178.608	4.418	12,999
Including emot. closeness	−0.369^***^ (0.132)	195.532	4.418	12,996
Including freq. contact	−0.469^***^ (0.162)	140.999	4.418	12,999
Including labor force status	−0.310^**^ (0.128)	209.655	4.429	11,701
Excluding income	−0.320^**^ (0.130)	194.716	4.417	13,866
Controls with replaced missings	−0.141 (0.098)	310.305	4.417	28,397
	*Reduced form effect*	$\beta_{\mathrm{lowerbound}}$	$\beta_{\mathrm{upperbound}}$	$\gamma_{\max}$
Relaxing exclusion restriction	−0.074** (0.030)	−0.566	0.026	−0.022
Placebo: Birth weight	156.638 (250.410)	111.613	3395.2	7842
Placebo: Low birth weight	−0.037 (0.096)	111.613	0.054	7842

*Note:* Robust standard errors clustered at the household level in parentheses. The general health variable is an ordinal variable on a scale from 1 (bad health) to 5 (good health). The regressions include the control variables listed in Table A1 column (a).

*Source:* Pairfam (2010–2020), weighted, own calculation.

^*^ *p* < 0.10.

^**^ *p* < 0.05.

^***^ *p* < 0.01.

**TABLE A14:** Balancing table.

	Distance
No/lower school degree	−0.0458
	(0.0381)
University degree	−0.161^***^
	(0.0272)
Migration background	−0.104^***^
	(0.0385)
log (income) in 1000€	−0.0395^*^
	(0.0237)
Age	−0.00575^*^
	(0.00332)
Children's sex	0.00571
	(0.0138)
Nr. children 0–2	0.0681^**^
	(0.0269)
Nr. children 3–5.5	0.0688^***^
	(0.0260)
Nr. children 5.5–10	0.0191
	(0.0169)
Nr. other children	0.000814
	(0.0170)
Grandparent's age	0.00124
	(0.00225)
Pregnant	0.0307
	(0.0235)
Cohabitation with partner	0.0338
	(0.0298)
Widowed	0.140
	(0.0862)
Mother is single child	0.0526^*^
	(0.0279)
No school degree (grandm.)	0.0366
	(0.0589)
Upper school degree (grandm.)	−0.0458
	(0.0337)
No school degree (grandf.)	−0.0570
	(0.0718)
Upper school degree (grandf.)	−0.0642^**^
	(0.0327)
Children < 6 in kita	−0.0441^**^
	(0.0174)
Age youngest child	0.000462
	(0.000388)
Observations	9259

*Note:* Estimated using OLS based on the sample used in the regressions for maternal satisfaction. Robust standard errors clustered at the household level in parentheses. Regression includes individual and household controls described in Table A1 column (c).

*Source:* Pairfam (2009–2020), own calculations.

^*^ *p* < 0.10.

^**^ *p* < 0.05.

^***^ *p* < 0.01.
